# Continental Drift and Speciation of the *Cryptococcus neoformans* and *Cryptococcus gattii* Species Complexes

**DOI:** 10.1128/mSphere.00103-17

**Published:** 2017-04-19

**Authors:** Arturo Casadevall, Joudeh B. Freij, Christopher Hann-Soden, John Taylor

**Affiliations:** a Department of Molecular Microbiology and Immunology, Johns Hopkins Bloomberg School of Public Health, Baltimore, Maryland, USA; b University of California, Berkeley, Berkeley, California, USA; Duke University Medical Center

**Keywords:** *Cryptococcus neoformans*, evolution, fungus

## Abstract

Genomic analysis has placed the origins of two human-pathogenic fungi, the *Cryptococcus gattii* species complex and the *Cryptococcus neoformans* species complex, in South America and Africa, respectively. Molecular clock calculations suggest that the two species separated ~80 to 100 million years ago. This time closely approximates the breakup of the supercontinent Pangea, which gave rise to South America and Africa. On the basis of the geographic distribution of these two species complexes and the coincidence of the evolutionary divergence and Pangea breakup times, we propose that a spatial separation caused by continental drift resulted in the emergence of the *C. gattii* and *C. neoformans* species complexes from a Pangean ancestor. We note that, despite the spatial and temporal separation that occurred approximately 100 million years ago, these two species complexes are morphologically similar, share virulence factors, and cause very similar diseases. Continuation of these phenotypic characteristics despite ancient separation suggests the maintenance of similar selection pressures throughout geologic ages.

## INTRODUCTION

Cryptococcosis is a disease of humans and animals that is caused by *Cryptococcus* spp. The disease is most frequent in individuals with impaired immunity, and there are currently over a million cases worldwide (1). In recent years, most pathogenic cryptococcal strains have been grouped within two species known as *Cryptococcus neoformans* and *C. gattii*, but genomic analysis reveals a complex taxonomy such that each of these taxa almost certainly includes numerous individual species (2). Given the rapidly accumulating genomic information and concerns about nomenclature instability, it was recently proposed that species complex nomenclature be used such that the broad taxa *C. neoformans* and *C. gattii* will be referred to as the *C. neoformans* species complex and the *C. gattii* species complex (3), a temporary expedient that we use in this essay. Each of these species complexes potentially includes numerous species, but those in each complex are more closely related to each other than to those across the two complexes.

The same genomic studies that have shown great taxonomic diversity have also provided important new insights into the evolution of the *C. gattii* and *C. neoformans* species complexes. The research effort launched to understand the origins of the *C. gattii* strains causing outbreaks among otherwise healthy humans in the Pacific Northwest of Canada and the United States revealed that the best-studied lineage of the *C. gattii* species complex has a center of genetic diversity that includes individuals of both the **a** and α mating types in the rainforest of Northern Brazil (4–6). Subsequent studies of additional Brazilian isolates suggest that an even more diverse population of the *C. gattii* species complex may be found in more arid areas of northwest Brazil (7). In contrast, in the *C. neoformans* species complex, the lineage known as VNB, or VNII-AFLP1A, has a center of genetic diversity that again includes individuals of both the **a** and α mating types in the Botswana region of southern Africa (8). Consistent with an African origin of *C. neoformans* var. *grubii* is an analysis of isolates in Southeast Asia that reveals low genetic diversity (9). Consequently, the available genomic data place the origins of lineages of the *C. gattii* and *C. neoformans* species complexes on two different continents. Previously, molecular clock calculations based on an estimated 32% substitution of synonymous nucleotide positions in orthologous coding regions and a neutral mutation rate of 2 × 10^−9^ substitutions per nucleotide per year suggested that the two cryptococcal species complexes separated about 80 million years ago (mya), with a range of 16 to 160 mya (10). We aligned 13.6 × 10^6^ nucleotides of the genomes of *C. neoformans* species complex *B-3501A* and *C. gattii* species complex* WM276*, finding that 15.54% were polymorphic, a value that grew to 17.4% after multiple substitutions were accounted for (11). Using the full range of mutation rates estimated for coding regions in ascomycete filamentous fungi (0.9 × 10^−9^ to 16.7 × 10^−9^) (12), the divergence time would lie between 5.2 ×10^6^ and 96.7 ×10^6^ years ago, which encompasses the previous estimate. If the recently published mutation rate measured experimentally in *Saccharomyces cerevisiae*, 1.6 × 10^−10^, were used, the divergence would be pushed back to 544 × 10^6^ years before the present, which seems far too long ago (13, 14). Realizing that no substitution rate has been estimated for *Basidiomycota* and cognizant of the strong difference between the substitution rates estimated for filamentous *Ascomycota* and yeast, we have no basis on which to dispute a divergence time of 80 × 10^6^ to 100 × 10^6^ years, which would lie roughly in the middle of the Cretaceous period (145 to 65 mya).

**FIG 1:**
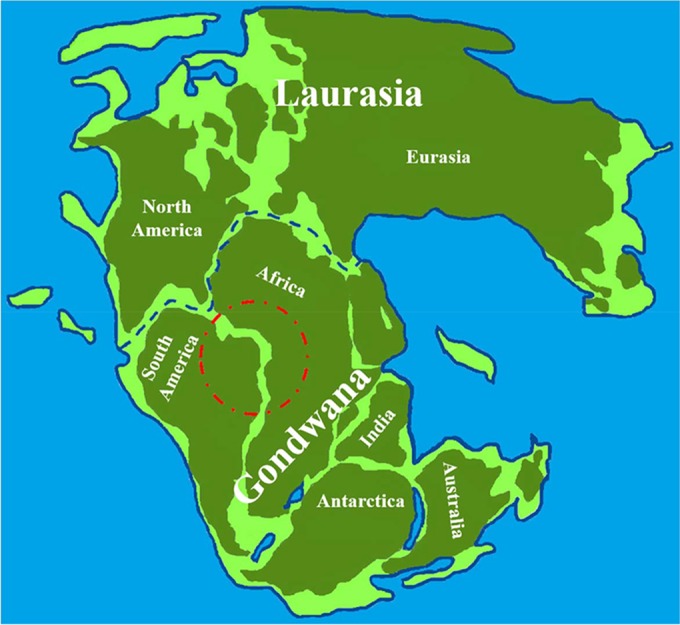
Representation of the supercontinent of Pangea with outlines of the present continents. The red circle denotes the proposed biogeography of the Pangean ancestor of both the *C. gattii* and *C. neoformans* species complexes. Also shown are the two supercontinents that came together to form Pangea, Laurasia and Gondwana (separated by a dashed blue line). This map was designed by tracing an outline of the supercontinent of Pangea via a Google Image search. The organization of subcontinents (Laurasia and Gondwana) is based on reference 47.

Reviewing the geography of planet Earth at the time that the cryptococcal species complexes separated places us at a geologic time dominated by the breakup of the supercontinent Pangea. This breakup formed the minor supercontinent of west Gondwana, which subsequently broke up to generate the current continents of South America and Africa. The breakup of west Gondwana began in the Early Cretaceous, about 130 mya, and Africa was separate from South America by approximately 100 mya. However, dinosaur fossil data suggest that some land connections between Africa and South America existed as late as 95 mya (15). The details of the breakup of west Gondwana leading to the formation of the South Atlantic are lost in time, but the process is thought to have occurred gradually over tens of millions of years. Although the time estimates for the divergence of the cryptococcal species complexes and the separation of the South American and African continents both have tremendous uncertainty, we are struck by the coincidence of the time scales of both processes and the ancient geographic juxtaposition of the regions now posited as regions of origin. On this basis, we hypothesize that the *C. gattii* and *C. neoformans* species complexes emerged following continental drift events that resulted in the physical separation of a Pangean cryptococcal ancestor population that occupied a discrete region of the supercontinent Pangea, a region that subsequently became parts of South America and Africa (Fig. 1). Given the modern association of *Cryptococcus* species with birds, particularly invasive rock doves (16), it might be thought that continental rafting would have scant effect on the distribution of the fungus given the potential for the aerial transport of fungal isolates across great distances. Although birds evolved from dinosaurs in the Early Cretaceous, the major radiation of modern birds occurred after the end of the Cretaceous at 65 mya, and on the basis of the phylogeny of birds, the radiation of the clade that includes the rock dove occurred after 25 mya (17). Nevertheless, it is possible that there was an association between the primordial *Cryptococcus* ancestor and early birds, which could have allowed the intercontinental transport of isolates during the early phases of the Pangean breakup, when the continents were much closer than they are today and thus prevented species isolation until more recent epochs. Such intercontinental transport of isolates by birds, wind, ocean currents, or even another animal would account for estimates of a more recent separation of the *Cryptococcus* complex species. One can imagine scenarios where intercontinental transport mechanisms prevented isolation of the primordial *Cryptococcus* ancestor for millions of years following continental separation until increasing continental distance created conditions for spatial isolation. Hence, the wide difference between estimates of the separation time of the *C. gattii* and *C. neoformans* species complexes, which include significantly more recent dates than the separation of Africa and South America, could still be reconciled with a precipitating event associated with the breakup of Pangea. Furthermore, we note that the suggested speciation event is very different from the current situation, where lineages of both complexes occur simultaneously in diverse geographic regions throughout the globe. We propose that continental drift was the initial trigger of speciation and that the current geographic distribution of the two cryptococcal species complexes is the result of subsequent introduction and dispersal events, including anthropomorphic causes, such as the global dispersal of *Columbia livia* (rock dove or pigeon) from its Mediterranean origin in recent centuries (16).

One of the remarkable aspects of the two cryptococcal species complexes is how similar they are with regard to virulence factors despite their distant separation in time. For example, the two cryptococcal complex species complexes share such virulence-associated phenotypes as polysaccharide capsules (18), thermotolerance of mammalian temperatures (18), melanin production (18), urease (19, 20) and phospholipase (21, 22) activities, intracellular replication (23, 24), nonlytic exocytosis (25, 26), and inositol production (27). Although it is possible that these phenotypes evolved independently, the fact that they are not shared, or rarely shared, by other closely related species supports the view that they evolved in the common ancestor of both the *C. neoformans* and *C. gattii* species complexes. For example, a survey of heterobasidiomycetous yeasts found that only *Cryptococcus podzolicus* had a capsule and was capable of making melanin (28). Both cryptococcal species complexes have been isolated from tree hollows (29), and we note that their current and past locations are near forested equatorial regions. Strains of both cryptococcal species complexes have been shown to be facultative intracellular pathogens capable of replicating in mammalian macrophages. For *C. neoformans*, the intracellular pathogenic strategy in macrophages and amoebae has been shown to be uncannily similar, which led to the proposal that the capacity for animal virulence arose from selection pressures in the environment that included predaceous phagocytic cells such as those now found in amoebae and slime molds (30, 31). Strains of both cryptococcal species complexes have been shown to interact with amoebae, which they can exploit for growth under certain circumstances (30, 32). Given that the origins of both fungi and amoebae occur in deep Earth time and that the closest outgroup to the kingdom Fungi is that of nucleariid amoebae (33), the ancestors of these two groups of eukaryotes could have been interacting even before the emergence of multicellular life forms. Consequently, we hypothesize that the remarkable similarities between the two cryptococcal species complexes with regard to their distinctive encapsulated morphology and shared virulence factors is a consequence of similar selection pressures, which continued after the breakup of West Gondwana.

The facts that the *C. neoformans* and *C. gattii* species complexes are likely to have diverged in the Cretaceous and share so many phenotypes associated with virulence have interesting implications for their Pangean ancestor and for the origin of virulence in fungi. First, it must have shared the virulence phenotypes now found in *C. gattii* and *C. neoformans*, which implies that it was encapsulated, made melanin, possessed numerous enzymes that can damage host cells, and had the capacity for intracellular replication in animal and environmental phagocytic cells. Second, since cryptococcosis can occur in reptiles (34) and the Cretaceous was remarkable for its reptilian megafauna, the Pangean ancestor could have been pathogenic for some animals at the time. Third, the capacity of cryptococcal species for virulence must have existed in the ancient past. Since fungal and amoebal evolutionary lineages predate the appearance of animals and interactions between these organisms, such as predation of fungi by amoebae, may have selected for traits that accidentally enabled a capacity for mammalian virulence, it is conceivable that fungi with the potential to be pathogenic in metazoans existed before the latter appeared. A thorough phylogenetic and phenotypic study of *C. gattii* and *C. neoformans* and close relatives in the *Filobasidella* and *Kwoniella* clades, many of which had been isolated from insect guts or frass, concluded that ancestors of the human pathogens had the abilities to grow at 30°C, produce a capsule, and produce melanin (35). It seems likely, therefore, that the ancestors of the pathogenic *Cryptococcus* species had the capacity for animal virulence, which was passed on to the descendant species. An origin of virulence in *Cryptococcus* complex species in deep time could help explain the remarkable nonspecificity of their pathogenic potential, given their capacity for virulence to vertebrates (18), insects (36, 37), nematodes (38), amoebae (30), and plants (39). This scenario differs from the situation with two groups of *Ascomycota*, *Onygenales* (40) and *Clavicipitales* (41), where virulence emerged independently. Interestingly, in the case of *Clavicipitales*, the animal-parasitic species may have evolved from mycoparasitic ancestors. Relatives of the pathogenic *Cryptococcus* species in the *Tremella* clade are also mycoparasitic, but it appears likely that this trait was not exhibited by the ancestor of the *C. gattii* and *C. neoformans* species complexes (35).

Coming back to continental drift and its ability to change the continuity of land masses and isolate species or bring them together, in addition to *Cryptococcus* species, this phenomenon has been used to explain the biogeography of many land species, including animals and plants and their associated microbes. Continental drift has been used to interpret the biogeography of many pathogenic microbes, including geminiviruses (42), trypanosomes (43), and the conifer root rot fungus *Heterobasidion annosum* (44). Here we extend that reasoning to the human-pathogenic cryptococcal species complexes and note with great interest the congruence of molecular and geologic scales and the glimpse they provide into the origins of fungal virulence. We are fully aware that we are just beginning to sample the genetic diversity of the fungal world and that as additional data are accrued, the current hypothesis may be further supported, require modification, or be abandoned. In associating cryptococcal speciation with continental drift, the most uncertain estimates are those of the divergence of the *C. neoformans* and *C. gattii* complex lineages. To improve the estimate of the time of divergence of the two *Cryptococcus* lineages would require either a measured mutation rate for *Cryptococcus* spp. or a solid geologic calibration for at least one node on a phylogenetic tree that includes the two *Cryptococcus* species, neither of which is available at the present time.

Our goal in formulating this hypothesis is to stimulate thought and discussion about the mechanisms driving fungal speciation and the origins of fungal virulence that will, we hope, promote further experimental work. In this regard, we note that the hypothesis already suggests new lines of inquiry. For example, if continental drift did indeed trigger the speciation of the *C. neoformans* and *C. gattii* complexes, then there may be a great level of spatial difference between individual cryptococcal lineages among different continents, which could be revealed by careful geographic environmental sampling. Also, the hypothesis suggests that the current distribution of *C. neoformans* and *C. gattii* complex strains should be viewed as resulting from a layering of strains descending from ancient ancestors with more recent dispersal events. In this regard, the occurrence of *C. neoformans* and *C. gattii* hybrids (45) could represent descendants of ancient mating events or recent crosses following the introduction of strains that retained the capacity for sexual reproduction. In this regard, the hypothesis provides a new conceptual approach to understanding their origin. For example, if these hybrids are the result of ancient mating events, these strains should be more prevalent in areas of Africa and South America that remained connected or physically related for the longest time. Finally, we hope that the hypothesis stimulates more research into the mutation rates of the cryptococcal complex species and into the nascent field of paleomycology. With regard to the latter, fungi from the Cretaceous have been found in amber (46) and a search for yeast related to *Cryptococcus* in amber combined with advanced DNA and protein sequencing techniques could provide important information to support, modify, or refute the proposed hypothesis.
